# Supplementary material for: The Ameliorative Effects of Fucoidan in Thioacetaide-Induced Liver Injury in Mice
Source: Molecules. 2021 Mar 30;26(7):1937. doi: 10.3390/molecules26071937 (PMC8036993; doi:10.3390/molecules26071937)
Supplement: Supplementary file 1 [file molecules-26-01937-s001.pdf]

Supplementary Materials

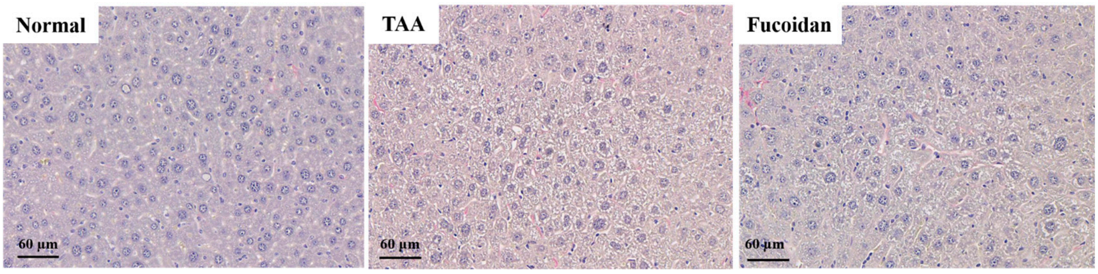

Figure 1. Changes of liver histology by sirius red staining (magnification, 200×).

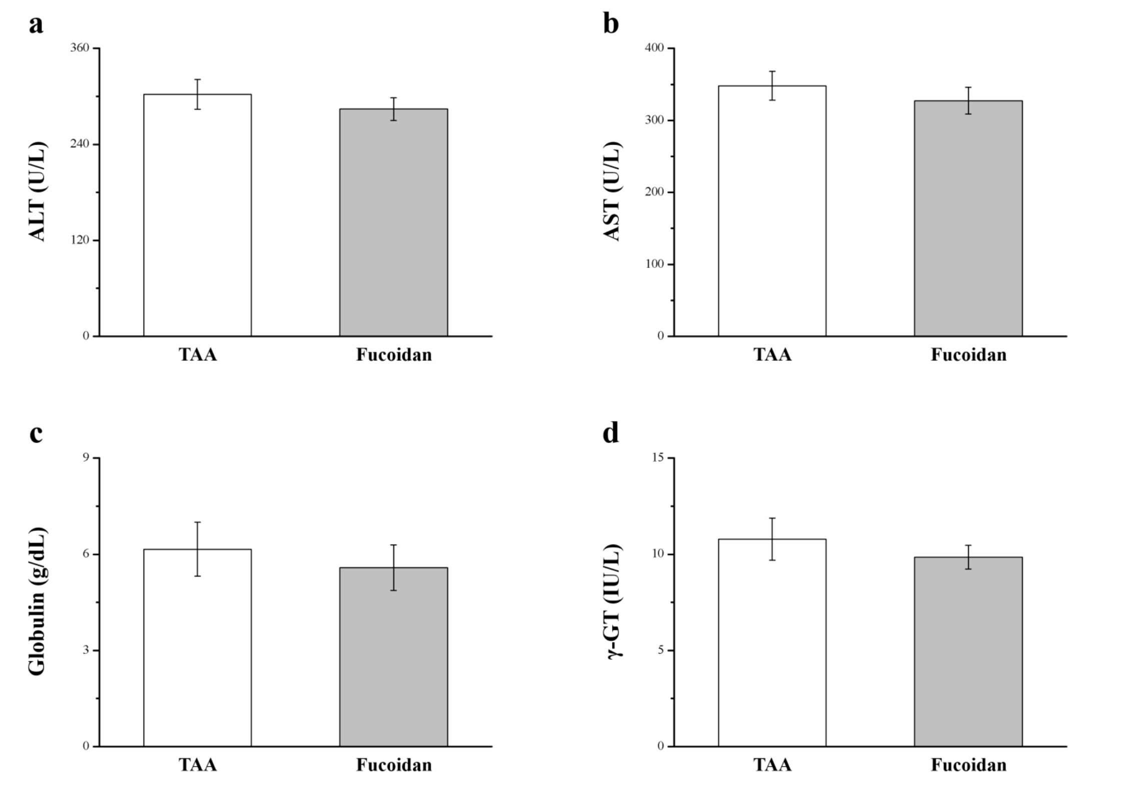

Figure 2. Changes of serum ALT, AST, globulin, and γ-GT levels in TAA-induced liver injury mice, and fucoidan (10 mg/kg)-treated mice with TAA-induced liver injury, over a period of 42 days.
